# A novel pathway to detect and cope with exogenous dsDNA

**DOI:** 10.1080/19420889.2015.1065361

**Published:** 2015-08-27

**Authors:** Shouhei Kobayashi, Tokuko Haraguchi

**Affiliations:** 1 Advanced ICT Research Institute Kobe, National Institute of Information and Communications Technology; Nishi-ku, Japan; 2 Graduate School of Frontier Biosciences, Osaka University; Suita, Japan; 3 Graduate School of Science, Osaka University; Toyonaka, Japan

**Keywords:** autophagy, barrier-to-autointegration factor, DNA sensor, endosome breakdown, exogenous DNA, live CLEM, nuclear envelope

## Abstract

How a living cell responds to exogenous materials is one of the fundamental questions in the life sciences. In particular, understanding the mechanisms by which a cell recognizes exogenous double-stranded DNA (dsDNA) is important for immunology research because it will facilitate the control of pathogen infections that entail the presence of exogenous dsDNA in the cytoplasm of host cells. Several cytosolic dsDNA sensor proteins that trigger innate immune responses have been identified and the downstream signaling pathways have been investigated. However, the events that occur at the site of exogenous dsDNA when it is exposed to the cytosol of the host cell remain unknown. Using dsDNA-coated polystyrene beads incorporated into living cells, we recently found that barrier-to-autointegration factor (BAF) binds to the exogenous dsDNA immediately after its appearance in the cytosol and plays a role in DNA avoidance of autophagy. Our findings reveal a novel pathway in which BAF plays a key role in the detection of and response to exogenous dsDNA.

The rapid detection of exogenous materials and subsequent responses are important for the survival of living cells in variable environments.^1^ A great deal of effort has been made to identify cellular factors that detect the invasion of exogenous dsDNA and mediate immune responses, such as type I interferon production.^2-11^ Downstream pathways after dsDNA detection have also been extensively investigated.^12-13^ Recently, stimulator of interferon genes (STING), which is an endoplasmic reticulum-associated protein that mediates type I interferon responses, has been identified as a critical factor in the regulation of an innate immune signaling pathway triggered by the invasion of exogenous dsDNA.^14^ However, cell biological information regarding when and where cytosolic dsDNA sensors detect exogenous dsDNA is lacking owing to the difficulties in monitoring exogenous dsDNA that enters the cytosol of cells.

Understanding intracellular fates of exogenous dsDNA after endosome breakdown is also important for the development of appropriate dsDNA carriers that ensure an efficient and yet safe nucleic acid delivery system. Many non-viral dsDNA carriers, including lipid-based and polymer-based DNA carriers, have been developed and are widely used for transgene expression.^15,16^ Most such dsDNA carriers were designed to induce efficient endosome breakdown because the endosome membrane is the first cellular barrier against transgene expression.^17^ However, even when free dsDNA is directly microinjected into the cytoplasm of a cell, only limited amount is effectively delivered to the nucleus,^18,19^ suggesting that other barriers to transgene expression exist in the cytoplasm. Because nuclear injection of dsDNAs is more effective than cytoplasmic injection for transgene expression from both non-viral dsDNA^19^ and cloned viral dsDNA,^20^ the nuclear envelope (NE) may act as a potent structural barrier for the delivery of dsDNA to the nucleus. Thus, understanding the fate of the exogenous dsDNA that invades the cytosol after endosome breakdown is important to achieve an efficient gene delivery system using non-viral carriers.

We recently reported the use of DNA-coated beads in which barrier-to-autointegration factor (BAF) acts as a cytosolic dsDNA sensor in mammalian cells.^21^ BAF, a highly conserved DNA binding protein, was first discovered as a cellular factor that prevents retroviral DNA from suicidal auto-integration and ensures its integration into host DNA.^22,23^ In addition to this virus-related function, BAF is known to function in host cell NE assembly^24,25^ and in S-phase progression.^26^ We found that when dsDNA-coated beads were incorporated into HeLa cells with a transfection reagent, BAF accumulated at the site of exogenous dsDNA immediately after its appearance in the cytosol at endosome breakdown (**Fig. 1A**). BAF also accumulated at the site of microinjected dsDNAs, independent of their forms (linearized, circularized, or complexed with a transfection reagent) (**Fig. 1C**). Our findings suggest that BAF is able to detect various forms of dsDNA in living cells, even when it forms a complex with other molecules. Our finding is consistent with those of previous reports indicating that BAF binds to dsDNA in a sequence-nonspecific manner *in vitro*.^27,28^ Interestingly, our experimental system using DNA beads revealed that the NE-like membrane started to assemble around dsDNA within several minutes after its detection by BAF, and enwrapped the dsDNA within another 10–15 min in a BAF-dependent manner (compare **Fig. 1A and B**). This BAF-mediated NE-like membrane assembly at the dsDNA protected the dsDNA from autophagy (**Fig. 1A**). This was the first direct evidence of intracellular membrane remodeling that is triggered at the site of exogenous dsDNA invasion.

**Figure 1.**
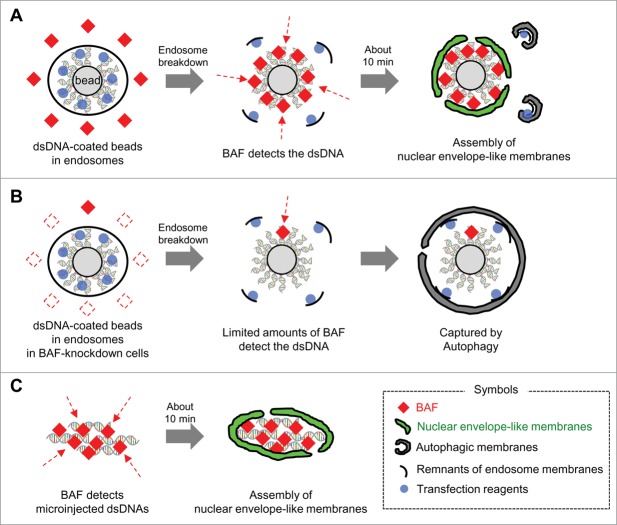
BAF-mediated pathway to detect and cope with exogenous dsDNA in mammalian cells. (**A**) When dsDNA-coated beads were incorporated into cells with transfection reagents, BAF detected exogenous dsDNA immediately after endosome breakdown around the beads, and then induced assembly of nuclear envelope (NE)-like membranes. This NE-like membrane assembly leads to the avoidance of exogenous dsDNA from autophagy that targets the remnants of endosome membranes. (**B**) Knockdown of BAF caused a significant decrease in the assembly of NE-like membranes and increased the formation of autophagic membranes around the DNA-beads. (**C**) When dsDNAs were microinjected into cells, BAF immediately accumulated at the site of injected dsDNA and induced NE-like membrane assembly at the region. Symbols are common in **Figs. 1A–C**.

The physiological significance of this BAF-mediated sequestration of exogenous dsDNA from autophagy remains unknown. Because autophagy is believed to act as an intracellular defense system that leads the targets to degradation, BAF acts in a somewhat opposite manner from its defense function. We speculate that BAF-mediated NE-like membrane assembly may function as a stronger defense system against exogenous dsDNAs than autophagy by rapidly compacting and isolating the DNA to prevent gene expression and replication. In fact, it is known that BAF can compact viral DNAs by bridging them *in vitro*,^27-29^ and that BAF inhibits viral DNA replication in vaccinia virus infections by binding to the DNA,^30^ supporting our idea. Another possibility is that BAF-mediated NE-like membrane assembly unexpectedly assists exogenous dsDNA, conferring protection from degradation, and this may allow for the exogenous dsDNA to be imported into the nucleus during cell cycle progression. Further studies are needed to understand this enigmatic behavior of BAF with respect to exogenous dsDNA, and to understand the relationship between BAF-mediated NE-like membrane assembly and autophagic membrane assembly.

In conclusion, direct visualization of BAF-mediated detection of exogenous dsDNA exposed to the cytosol of host cells shows an intimate link between the detection of exogenous dsDNA and subsequent intracellular responses. Our bead-mediated method will provide new insight into such responses to the entry of exogenous materials into a cell.
